# mTAGs: taxonomic profiling using degenerate consensus reference sequences of ribosomal RNA genes

**DOI:** 10.1093/bioinformatics/btab465

**Published:** 2021-07-13

**Authors:** Guillem Salazar, Hans-Joachim Ruscheweyh, Falk Hildebrand, Silvia G Acinas, Shinichi Sunagawa

**Affiliations:** Department of Biology, Institute of Microbiology and Swiss Institute of Bioinformatics, ETH Zürich, 8093 Zürich, Switzerland; Department of Biology, Institute of Microbiology and Swiss Institute of Bioinformatics, ETH Zürich, 8093 Zürich, Switzerland; Department of Gut Microbes and Health, Quadram Institute Bioscience, NR4 7UQ Norwich, UK; Department of Digital Biology, Earlham Institute, NR4 7UZ Norwich, UK; Department of Marine Biology and Oceanography, Institute of Marine Sciences (ICM)-CSIC, 08003 Barcelona, Spain; Department of Biology, Institute of Microbiology and Swiss Institute of Bioinformatics, ETH Zürich, 8093 Zürich, Switzerland

## Abstract

Profiling the taxonomic composition of microbial communities commonly involves the classification of ribosomal RNA gene fragments. As a trade-off to maintain high classification accuracy, existing tools are typically limited to the genus level. Here, we present mTAGs, a taxonomic profiling tool that implements the alignment of metagenomic sequencing reads to degenerate consensus reference sequences of small subunit ribosomal RNA genes. It uses DNA fragments, that is, paired-end sequencing reads, as count units and provides relative abundance profiles at multiple taxonomic ranks, including operational taxonomic units based on a 97% sequence identity cutoff. At the genus rank, mTAGs outperformed other tools across several metrics, such as the F_1_ score by >11% across data from different environments, and achieved competitive (F_1_ score) or better results (Bray–Curtis dissimilarity) at the sub-genus level.

**Availability and implementation:**

The software tool mTAGs is implemented in Python. The source code and binaries are freely available (https://github.com/SushiLab/mTAGs). The data underlying this article are available in Zenodo, at https://doi.org/10.5281/zenodo.4352762.

**Supplementary information:**

Supplementary data are available at *Bioinformatics* online.

## 1 Introduction

The relative abundance of taxa in a microbial community can be estimated by classifying sequences of phylogenetic marker genes. A common approach involves the generation of polymerase chain reaction (PCR)-derived amplicon sequences using oligonucleotide primers to target highly conserved regions of the small subunit ribosomal RNA (SSU-rRNA) gene. However, this approach has several limitations due to the introduction of errors (Acinas *et al.*, 2005) and taxonomic selection biases (Hong *et al.*, 2009) in the PCR step, and the inconsistency of results when targeting different variable regions of theSU-rRNA gene (Claesson *et al.*, 2010). As an alternative, the generation of metagenomic data, i.e. by shotgun-sequencing of microbial community DNA, allows for an unbiased extraction of SSU-rRNA gene fragments (Logares *et al.*, 2014) and their subsequent classification to generate taxonomic profiles. However, current tools performing SSU rRNA gene-based taxonomic profiling of metagenomes (Bengtsson-Palme *et al.*, 2015; Guo *et al.*, 2016; Shah *et al.*, 2011; Xie *et al.*, 2016) suffer from shortcomings, such as their inability to use reads originating from any region of the SSU-rRNA gene (Bengtsson-Palme *et al.*, 2015; Guo *et al.*, 2016; Xie *et al.*, 2016) or a limitation of the taxonomic resolution to the genus rank (Bengtsson-Palme *et al.*, 2015; Shah *et al.*, 2011; Xie *et al.*, 2016).

The classification performance of SSU-rRNA gene fragments of PCR-targeted or metagenomic origin differs between tools using reference sequence databases of reduced complexity (e.g. Bolyen *et al.*, 2019; Matias Rodrigues *et al.*, 2017; Schloss *et al.*, 2009). The construction of such reference databases may thus be a critical factor, in particular at high taxonomic resolution, that is, at ranks below the genus level, such as the operational taxonomic unit (OTU) defined at a 97% sequence identity cutoff. Here, we tested if the use of the International Union of Pure and Applied Chemistry (IUPAC) code for nucleotides to generate a reference database, in which each OTU is represented by a degenerate consensus sequence of all respective members, would increase the accuracy of individual SSU-rRNA sequence classification and community composition profiling at different taxonomic ranks. We implemented this approach in a new taxonomic profiler for metagenomes named mTAGs. We show an advantage of this method over simply using the longest sequence as an OTU representative, and that at the genus level, mTAGs provides higher accuracy compared to other tools that are commonly used to classify SSU-rRNA gene fragments (Bolyen *et al.*, 2019; Caporaso *et al.*, 2010; Matias Rodrigues *et al.*, 2017; Schloss *et al.*, 2009).

## 2 Tool description

The mTAGs tool uses a reference database, which was built by first clustering sequences into OTUs within each genus defined in the full-length SILVA SSU database version 138 (Quast *et al.*, 2013) at 97% identity. Then, for each OTU a degenerate consensus sequence was generated using the IUPAC DNA code to represent all respective member sequences (see Supplementary Information). The tool is capable of processing single-end and pair-end reads, takes advantage of the information contained in any region of the SSU-rRNA gene and provides relative abundance profiles at multiple taxonomic ranks, including OTUs. mTAGs takes shotgun-sequenced metagenomic data as an input and uses hidden Markov models to detect sequence fragments from any position of the SSU rRNA gene. These fragments are aligned to the reference database and conservatively classified to a taxonomic rank (according to the SILVA taxonomy) by determining the last common ancestor of all target sequences. The runtime of mTAGs increases linearly with the size of the metagenome (see Supplementary Information) at a rate of 53 s per million reads processed (wallclock time using eight CPU threads; 306 s in CPU time) allowing the processing of deeply sequenced metagenomes in reasonable time (i.e. ∼100 million paired-end reads in ∼1.5 h). Although the primary use of mTAGs is the taxonomic profiling of metagenomes, it can also be used for profiling SSU-rRNA amplicon data or for classifying amplicon sequence variants produced by other methods (Callahan *et al.*, 2016; Edgar, 2016).

## 3 Results

**Fig. 1.**
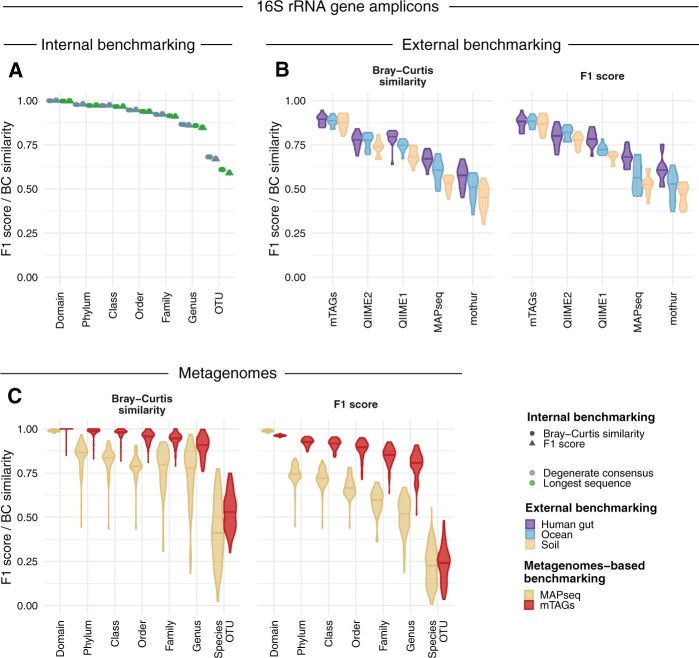
Benchmarking results on taxonomic profiling of microbial communities. (**A**) Internal benchmarking: benchmarking of the mTAGs reference database construction for read length of 150 bp. Values correspond to the performance in classification (F_1_ score) and profiling (Bray–Curtis similarity to the expected composition) at seven taxonomic ranks for the definition of the OTU representative sequence as (i) the degenerate consensus sequence of all respective members (blue) or (ii) the longest member sequence (green).The values of 10 independent evaluations are plotted. See the Supplementary Figure S1 for precision and recall values and results based on alternative read lengths. (**B**) External benchmarking: benchmarking of mTAGs against QIIME 1, QIIME 2, mothur and MAPseq using simulated datasets comprising the most abundant genera found in the human gut, ocean and soil environments (Almeida et al., 2018). Bray–Curtis similarity to the expected composition and F_1_ score values correspond to classifications at the genus-level (the lowest taxonomic rank common to all tools). To ensure comparability between the tools, the results are based on the SILVA SSU database version 128. See the Supplementary Information for more details and Supplementary Figure S2 for precision and recall values and results based on alternative reference databases. (**C**) Metagenomes-based benchmarking: benchmarking of mTAGs and MAPseq using metagenomic data from the second CAMI challenge (Meyer *et al.*, 2021). Values correspond to the performance in classification (F_1_ score) and profiling (Bray–Curtis dissimilarity to the expected composition) at seven taxonomic ranks

We benchmarked the effect of differences in the generation of the reference database by classifying reads of known identity (Fig. 1A;  Supplementary Fig. S1; Supplementary Information). The definition of the representative sequence for each OTU as a degenerate consensus sequence of all its respective members, rather than the longest sequence, resulted in a ∼14% increase in classification performance at the OTU level when profiling paired-end reads of 250 bp (14.0%, 14.1% and 14.0% for precision, recall and F_1_ score, respectively). A 25.4% increase in taxonomic profiling performance was observed as measured by an increase in the median Bray–Curtis similarity to the true profiles from 0.355 to 0.265 (Supplementary Fig. S1). This effect was still observed for reads of 150 bp, while no effect was found for reads of 100 bp and/or higher taxonomic ranks (Supplementary Fig. S1).

For an independent evaluation and comparison of classification and profiling performance, we used simulated data from previous work (Almeida *et al.*, 2018) using SSU-rRNA datasets comprising the most abundant genera found in the human gut, ocean and soil environments (Fig. 1B;  Supplementary Fig. S2) to benchmark a number of taxonomic profiling tools. In this comparison (Fig. 1B), mTAGs achieved a median F_1_ score of 0.88 and a median Bray–Curtis similarity to the expected abundance profile of 0.89 outperforming other tools classifying SSU-rRNA gene fragments down to the genus-level, the lowest taxonomic rank common to all tools (QIIME 1, QIIME 2, mothur and MAPseq achieved median F_1_ scores of 0.72, 0.80, 0.53 and 0.60 and Bray–Curtis similarities of 0.75, 0.77, 0.51 and 0.60, respectively). mTAGs had a high median precision of 0.98, comparable to the precision of MAPseq, and a median recall of 0.80, which was the highest value among the tested tools (Fig. 1B). This high classification performance was consistent for data from different environments (human gut, ocean and soil) and also when tested separately for different hyper-variable regions within the full-length SSU-rRNA gene (see Supplementary Information and Supplementary Fig. S2).

To assess the performance of mTAGs for shotgun metagenomics data and at the sub-genus level, a third evaluation was performed with human and mouse-associated metagenomes (Meyer *et al.*, 2021). This benchmark was performed in comparison with MAPseq, which was the only tool that provided outputs at the sub-genus taxonomic level (Fig. 1C; Supplementary Fig. S3). At this level (OTU level and NCBI species level for mTAGs and MAPseq, respectively) mTAGs achieved higher median Bray–Curtis similarity to the expected abundance profile, while the median F_1_ score was comparable between the tools (Fig. 1C). A breakdown of the F_1_ score showed a lower precision, but higher recall of mTAGs compared to MAPseq (Supplementary Fig. S3).

## 4 Conclusions

With mTAGs, we introduce a freely available tool for SSU-rRNA gene-based microbial community profiling that defines degenerate consensus sequences and uses them as a reference database to enable OTU-level relative abundance estimation.

## Supplementary Material

btab465_Supplementary_Data
