# Supplementary material for: mTAGs: taxonomic profiling using degenerate consensus reference sequences of ribosomal RNA genes
Source: Bioinformatics. 2021 Jul 13;38(1):270–2. doi: 10.1093/bioinformatics/btab465 (PMC8696115; doi:10.1093/bioinformatics/btab465)
Supplement: btab465_Supplementary_Data [file btab465_supplementary_data.zip › Salazar_Ruscheweyh_et_al_suppInfo.pdf]

Supplementary Information

Online Methods

Reference database and taxonomy

The non-redundant SILVA (v.138) database of publicly available full-length small subunit ribosomal RNA sequences (available at https://www.arb-silva.de/fileadmin/silva_databases/release_138/Exports/SILVA_138_SSURef_NR99_tax_silva_full_align_trunc.fasta.gz) was used as the starting point to build the mTAGs reference database. In order to define a new OTU-level taxonomic rank, fully consistent with the SILVA taxonomy, all-versus-all identities between sequences belonging to the same genus were computed and used to cluster them at 97% identity through complete linkage clustering using a python script. Sequences without genus annotation were clustered at their respective taxonomic level. The representative sequence for each cluster (that is, for each OTU) was defined as the degenerate consensus sequence, using the IUPAC DNA alphabet, of all its member sequences based on the above-mentioned alignment. Positions that were composed of >90% gaps were removed from the consensus sequence. A second reference database was built only for benchmarking purposes (and thus not released in the final version of the mTAGs tool) by taking the longest sequence in a cluster as its representative - the default behaviour in other clustering algorithms (Edgar 2010; Ghodsi, Liu, and Pop 2011; Rognes et al. 2016). A version of the mTAGs reference database was also built using the SILVA v.128 database for benchmarking purposes (see below).

The taxonomy from the original SILVA database was parsed to produce a standardized taxonomic affiliation for each cluster representative sequence comprising eight taxonomic ranks (root, domain, phylum, class, order, family, genus and OTU). Thus, all levels, except for root and OTU correspond to the original SILVA taxonomy. The root level was added to group all domains, while the OTU-level entries represent the taxonomic units generated through the clustering. The reference database is distributed with the tool as part of the installation process. The final database contains 233,519 reference sequences and the reference taxonomy comprises 1 root, 3 domains, 240 phyla, 696 classes, 1,715 orders, 2,809 families, 8,770 genera and 233,519 OTUs.

mTAGs pipeline

The mTAGs pipeline is a multi-threaded open-source software written in Python (v 3.7) freely available at https://github.com/SushiLab/mTAGs. It consists of two main steps for the extraction and annotation of rRNA sequences from metagenomes. The first step uses hmmer (Eddy 2011) for the detection of SSU-rRNA gene sequences using pre-computed hidden Markov models (available at https://github.com/SushiLab/mTAGs/tree/master/data). Insert sequences (*i.e.*, read pairs) are profiled using hidden Markov models for both the SSU-rRNA and LSU-rRNA gene to remove false positives. All inserts detected only with the SSU model are saved as FASTA files. If inserts are detected with both the SSU and LSU models, cases with the lower E-value are selected. An E-value cutoff of 0.01 is used in both cases. The second step performs the taxonomic annotation of these sequences through the alignment against the reference mTAGs database. Global pairwise alignments are performed using the USEARCH algorithm as implemented in vsearch (Rognes et al. 2016) with a minimum percentage identity of 97%. The heuristic nature of the alignment process,

which determines the trade-off between speed and sensitivity, can be controlled by the user through the definition of the termination options (*maxaccepts* and *maxrejects* parameters of the USEARCH algorithm). For each insert sequence, all alignments with the maximum alignment score (defined as 2· matches - 4 · (gaps + mismatches)) are further considered for taxonomic annotation. This is achieved by using a last common ancestor approach in which each insert sequence is assigned to a specific taxonomic rank if this is common to all the reference sequences detected through the alignment process. Finally, abundance profiles for each of the eight taxonomic ranks are computed by counting the number of insert sequences assigned to each taxonomic rank and the number of unaligned and unassigned insert sequences. The mTAGs software also provides a command for the merging of individual profiles into a single abundance table for downstream analyses when more than one metagenomic sample is processed.

Validation and benchmarking datasets

A first benchmarking exercise, *internal benchmarking* hereafter, aimed to evaluate the effect of the reference database construction, specifically the definition of the OTU representative sequence as the degenerate consensus sequence of all cluster members compared to the use of the longest sequence (see above). This was achieved through the classification of reads of known identity simulated from the same initial database used to build the mTAGs reference database. For each sequence in the SILVA SSU v.138 database, 100 inserts were randomly simulated in pair-end mode with read lengths of 100, 150 and 250 bp and a normally distributed insert size (mean of 350 bp), producing an initial benchmarking dataset with more than 150 million inserts. For each read length, 10 random samples of 100k inserts each were taken from this initial collection of simulated reads and used to evaluate the performance of mTAGs on the two reference databases described above. This strategy allowed us to fully evaluate the classification accuracy as the taxonomic affiliation of each simulated insert is known.

A second benchmarking exercise, *external benchmarking* hereafter, was conducted with the aim of comparing the classification accuracy of mTAGs to that of competing taxonomic classifiers of 16S rRNA amplicons. For this purpose, a benchmarking dataset which is independent of the reference database used by the tool is desired. We thus made use of a pre-existing collection of standardized benchmarking datasets (Almeida et al. 2018) comprising the most abundant genera found in the human gut, ocean, and soil environments (available at ftp://ftp.ebi.ac.uk/pub/databases/metagenomics/taxon_benchmarking/), which have been used in previous benchmarking studies (Almeida et al. 2018; Lu and Salzberg 2020). This consists of a collection of datasets of pair-end reads of 250 bp simulated from the SSU-rRNA genes from the 80 most abundant genera in the human gut, ocean and soil environments. They comprise data simulated for several variable regions of the 16S rRNA gene (V1-2, V3-4, V4 and V4-5) with two levels of sequence depth (10k and 200k pair-end reads) and diversity (100 or 500 species simulated) and random mutations in 2% of the sequence positions. The collection also includes the results of benchmarking the taxonomic classifiers QIIME 1 v.1.9.1 (Caporaso et al. 2010), QIIME 2 v2017.11 (Bolyen et al. 2019), mothur v1.39.5 (Schloss et al. 2009) and MAPseq v1.2.2 (Matias Rodrigues et al. 2017) with the released datasets, allowing thus the comparison of any other classifier without the need of repeating this exercise for all tools. We took advantage of this and benchmarked mTAGs against these competing taxonomic classifiers by running the benchmarking datasets through the mTAGs pipeline and comparing the results to the ones provided for the competing tools as part of the collection. For better comparability, a reference database for mTAGs was built based on SILVA v.128 for this specific analysis as this was the version used to produce the

benchmarking collection in Almeida et al (2018). The data from the V1-V2 region was excluded from the analysis due to its low coverage in the databases, as recognised in the original publication.

A third benchmarking exercise, *metagenomes-based benchmarking* hereafter, was conducted with the dual goal of assessing the performance of mTAGs with metagenomic data and of comparing its classification accuracy below the genus level to that of MAPseq (the only tool of those described above capable of profiling below the genus level and using metagenomic data). Sequencing reads from 113 samples (toy datasets, human=49, mouse=64) generated for the second CAMI challenge (Meyer et al. 2021) were downloaded and profiled using mTAGs and MAPSeq (v1.2.6) with default parameters. As MAPseq uses the same taxonomy as the one provided by the CAMI dataset (NCBI taxonomy), a direct comparison of the profiles produced by MAPseq and the ground truth provided in the benchmarking dataset was straightforward. However, mTAGs is based on the SILVA taxonomy and thus the correct assignment of each read within the SILVA taxonomy needed to be established in order to perform the benchmarking. For this purpose the 2,612 genomes that were used to generate the simulated sequencing reads for the CAMI dataset were downloaded (https://data.cami-challenge.org/participate) and the *checkm ssu_finder* (Parks et al. 2015) command was used to extract the full-length 16S/18S gene sequences from the genomes. The 11,643 16S/18S gene sequences were then aligned against the current mTAGs database (v138, degenerate consensus) using vsearch with exhaustive search (with the command *vsearch --usearch_global --id 0.0 --maxaccepts 0 --maxrejects 0 --output_no_hits --strand both*). Alignments with an alignment coverage < 90% and a percent identity < 97% were discarded and the best alignment was picked based on percent identity to establish a map between genomes used in CAMI and OTUs in the degenerate mTAGs database. This map allowed us to assign each read at all taxonomic ranks used by mTAGs and thus to evaluate the performance of mTAGs using the CAMI dataset despite the discrepancy in the underlying taxonomy. The taxonomic rank below the genus level at which the two tools are able to profile is also different: mTAGs profiles at the OTU level (defined as 97% identity clusters) while MAPseq profiles at the species level (as defined in the NCBI taxonomy). For better comparability of the tools, the OTU-level profiles produced with mTAGs were translated into species level profiles by producing a map between OTUs and species using the NCBI taxonomy map released by SILVA (https://www.arb-silva.de/fileadmin/silva_databases/release_138/Exports/taxonomy/taxmap_ncbi_ssu_ref_138.txt.gz). Conservatively, an OTU was assigned to an NCBI species only if all original members shared the same species-level assignment. This procedure allowed for the evaluation of both tools at the same taxonomic rank (NCBI species level). However, it should be noted that this conservative procedure reduced the recall of mTAGs, since not all OTUs could be unambiguously assigned to an NCBI species.

Validation and benchmarking metrics

The assessment of the taxonomic profiling accuracy for mTAGs and the competing tools in all benchmarking exercises was done by computing the precision (or specificity), recall (or sensitivity), F_1 score and the Bray-Curtis similarity to the true profile (that is, the similarity between the abundance profile produced by each tool and the expected abundance profile). For the external benchmarking, we computed these metrics for QIIME 1, QIIME 2, mothur and MAPseq based on the abundance profiles released by Almeida et al (2018). For the metagenomes-based benchmarking, profiles were generated by running MAPseq and mTAGs using the CAMI dataset. As mTAGs is the only tool providing per-insert taxonomic assignments, precision, recall and F_1 score were also

computed based on insert annotations. This provides exact values for all three metrics, while profile-based computations might not account for cross-misclassification (see below).

If per-insert taxonomic annotations are available (as in the case of mTAGs), each insert can be defined as correctly assigned, incorrectly assigned or unassigned at each taxonomic level, by comparing the predicted and expected taxonomic annotation. Thus, the total number of correctly assigned (C), incorrectly assigned (I) and unassigned (U) inserts can be exactly computed. The precision corresponds to the fraction of correctly assigned inserts out of all assigned inserts. The recall is computed as the fraction of correctly assigned inserts out of all inserts (i.e. assigned and unassigned). Finally, the F_1 score is computed as the harmonic mean of precision and recall. The equations used for the computation were the following:

$$precision = \frac{C}{C + I}$$

$$recall = \frac{C}{C + I + U}$$

$$F1\,score = 2 \times \frac{precision \times recall}{precision + recall}$$

The number of correctly assigned, incorrectly assigned and unassigned inserts can be approximated when only the abundance profiles are available (as in the case of QIIME 1, QIIME 2, mothur and MAPseq). Given the expected abundance of taxon *i* (E_i) and the abundance predicted for this same taxon by any of the tools (P_i), the number of correctly assigned inserts (C_i) correspond to the minimum of these two values and the number of incorrectly assigned inserts (I_i) corresponds to the excess of P_i compared to E_i. Thus, the total number of correctly assigned inserts (C) and incorrectly assigned inserts (I) correspond to the sum of all C_i and I_i values for all taxa, respectively. Finally, the number of unassigned inserts (U) corresponds to the difference of the sum of the expected and predicted profiles. The precision, recall and F_1 score can then be computed with the equations described above. It is important to note that the metrics computed from the abundance profiles will only equal the values computed from the insert annotations if no cross-misclassifications exist. If a number of inserts from a given taxon *k* are assigned to taxon *l* and a number of inserts from taxon *l* are assigned to taxon *k*, only the excess of inserts in one taxon will be detected as incorrectly assigned inserts. Thus, depending on the frequency of cross-misclassifications precision, recall and F_1 score computed from abundance profiles might provide slightly higher values (but never lower) than the ones computed from insert annotations. This is apparent when precision, recall and F_1 score values computed from abundance profiles and from insert annotations are compared for mTAGs (Supplementary Figure 4). As the purpose of the present work is to benchmark mTAGs against competing tools, we based all analyses on the metrics computed for mTAGs on insert annotations, which provides exact values and which would, if anything, favor the competing tools.

Computation speed

The assessment of computation speed was performed by running mTAGs (default parameters) with 8 threads using a set of 370 deeply sequenced marine microbial metagenomes from the *Tara* Oceans Expedition (Salazar et al. 2019), which are accessible through ENA at https://www.ebi.ac.uk/ena using the identifiers listed in

https://doi.org/10.5281/zenodo.3473199. Wallclock and CPU time was tracked for each metagenome independently and related to the total number of inserts in each sample through a linear regression (Supplementary Figure 5).

All analyses were run in R statistical software (v.4.0.0) (R Core Team 2020). The scripts used for all analyses are available at https://doi.org/10.5281/zenodo.4352762.

References

Almeida,A. *et al.* (2018) Benchmarking taxonomic assignments based on 16S rRNA gene profiling of the microbiota from commonly sampled environments. *Gigascience*, **7**.

Bolyen,E. *et al.* (2019) Reproducible, interactive, scalable and extensible microbiome data science using QIIME 2. *Nat. Biotechnol.*, **37**, 852–857.

Caporaso,J.G. *et al.* (2010) QIIME allows analysis of high-throughput community sequencing data. *Nat. Methods*, **7**, 335–336.

Eddy,S.R. (2011) Accelerated Profile HMM Searches. *PLoS Comput. Biol.*, **7**, e1002195.

Edgar,R.C. (2010) Search and clustering orders of magnitude faster than BLAST. *Bioinformatics*, **26**, 2460–2461.

Ghodsi,M. *et al.* (2011) DNACLUST: accurate and efficient clustering of phylogenetic marker genes. *BMC Bioinformatics*, **12**, 271.

Lu,J. and Salzberg,S.L. (2020) Ultrafast and accurate 16S rRNA microbial community analysis using Kraken 2. *Microbiome*, **8**, 124.

Matias Rodrigues,J.F. *et al.* (2017) MAPseq: highly efficient k-mer search with confidence estimates, for rRNA sequence analysis. *Bioinformatics*, **33**, 3808–3810.

Meyer,F. *et al.* (2021) Tutorial: assessing metagenomics software with the CAMI benchmarking toolkit. *Nat. Protoc.*, **16**, 1785–1801.

Parks,D.H. *et al.* (2015) CheckM: assessing the quality of microbial genomes recovered from isolates, single cells, and metagenomes. *Genome Res.*, **25**, 1043–1055.

R Core Team (2020) R: A Language and Environment for Statistical Computing.

Rognes,T. *et al.* (2016) VSEARCH: a versatile open source tool for metagenomics. *PeerJ*, **4**, e2584.

Salazar,G. *et al.* (2019) Gene Expression Changes and Community Turnover Differentially Shape the Global Ocean Metatranscriptome. *Cell*, **179**, 1068–1083.e21.

Schloss,P.D. *et al.* (2009) Introducing mothur: open-source, platform-independent, community-supported software for describing and comparing microbial communities. *Appl. Environ. Microbiol.*, **75**, 7537–7541.

Supplementary figures

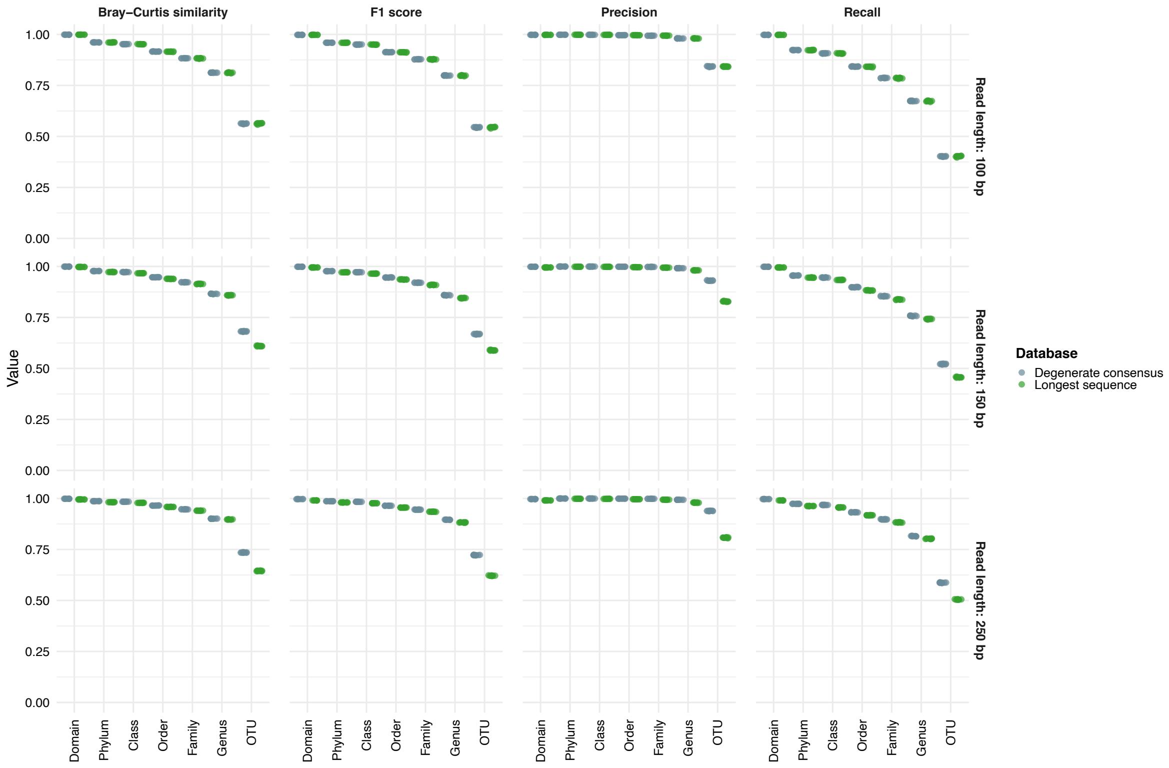

Supplementary Figure 1: Results of the internal benchmarking for read lengths of 100, 150 and 250 bp and including all evaluation metrics (precision, recall, F_1 score and Bray-Curtis similarity to the expected composition).

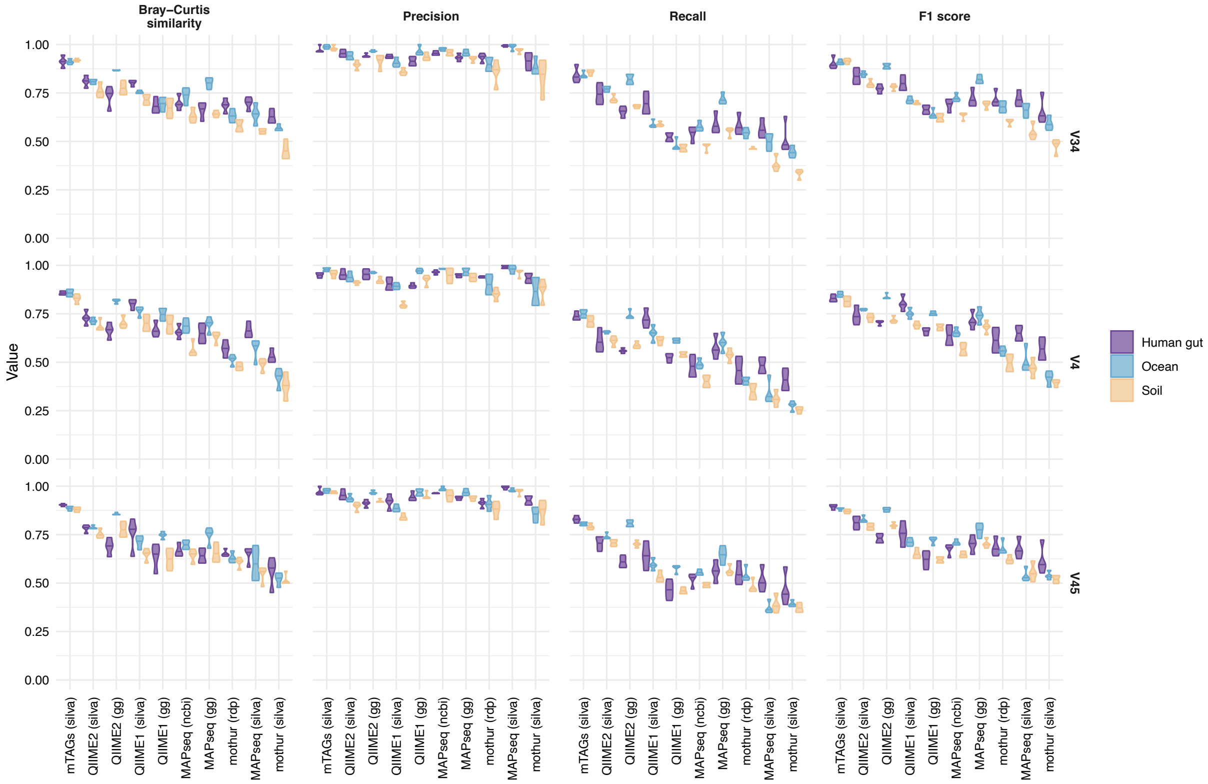

Supplementary Figure 2: Results of the external benchmarking including all evaluation metrics (precision, recall, F_1 score and Bray-Curtis similarity to the expected composition), alternative databases for the competing tools (silva: SILVA database, gg: Greengenes database, rdp: RDP database, ncbi: NCBI database) and separate results for the V3-V4, V4 and V4-V5 regions of the 16S rRNA gene. See Almeida et al. (2018) for details.

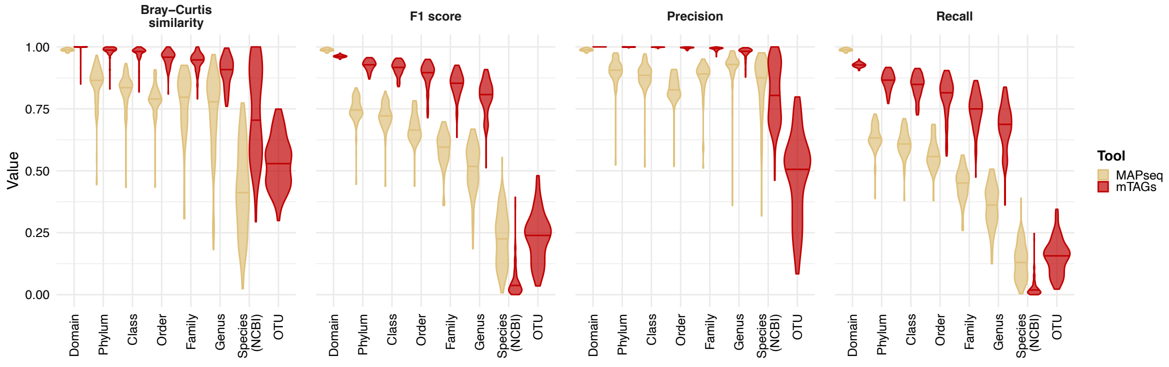

Supplementary Figure 3: Results for the metagenomes-based benchmarking including all evaluation metrics (precision, recall, F_1 score and Bray-Curtis similarity to the expected composition). mTAGs generally outperforms MAPseq across all taxonomic levels down to the genus level. When comparing the tools at their lowest taxonomic rank (NCBI species and OTUs for MAPseq and mTAGs, respectively), mTAGs showed a higher Bray-Curtis similarity, and a higher recall, but lower precision, resulting in a similar F_1 score. For a comparison at the same taxonomic rank, mTAGs OTUs were assigned to NCBI species; however, only if all member sequences were annotated the same NCBI species. This resulted in an increase in precision but a decrease in recall due to the lack of consistency between SILVA and NCBI taxonomies (see Supplementary Information).

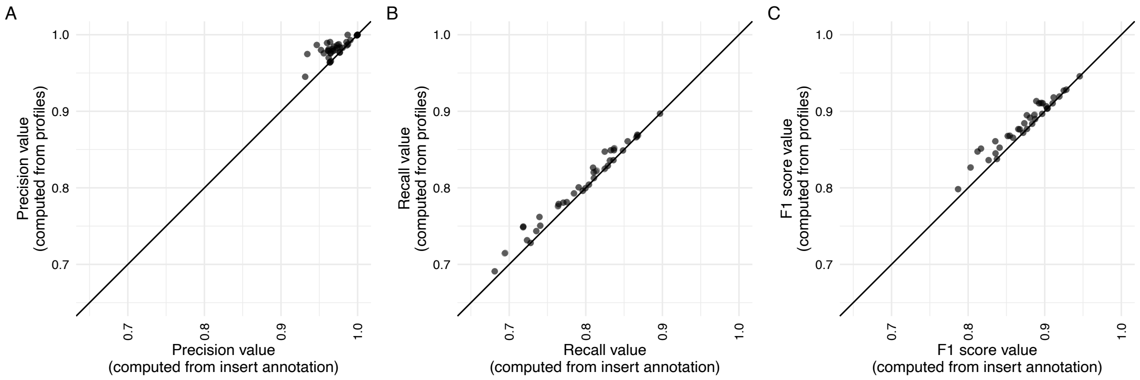

Supplementary Figure 4: Comparison between the (A) precision, (B) recall and (C) F_1 score computed from the abundance profiles (x-axis) and from the insert annotations (y-axis) for genus-level classification using the data for mTAGs in the external benchmarking (*i.e.*, same data as in Figure 1B).

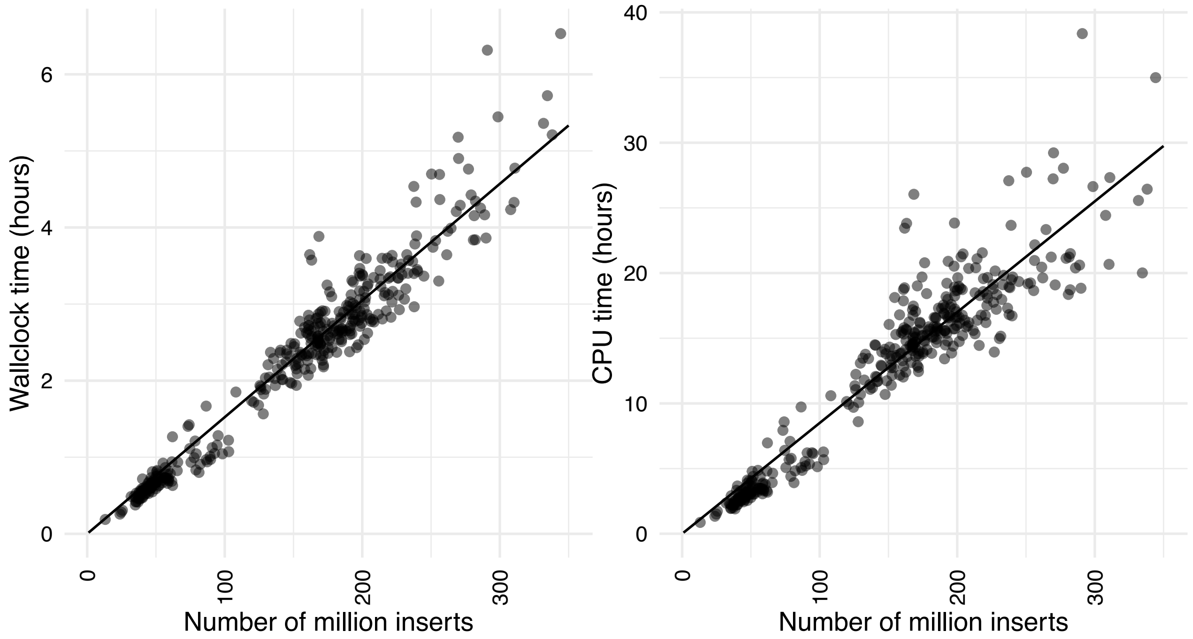

Supplementary Figure 5: Runtime of mTAGs as a function of the metagenome size. Wallclock time (left) and CPU time (right) in relation to the total number of inserts when executing mTAGs with default parameters for 370 marine metagenomes from the *Tara* Oceans Expedition using up to 8 CPU threads. The line corresponds to the best least-square linear regression through the origin (left: $y = 0.001524 \cdot x$, $R^2 = 0.986$; right: $y = 0.085 \cdot x$, $R^2 = 0.973$).

Supplementary File 1: Performance statistics (precision, recall, F_1 score and Bray-Curtis similarity to the expected composition) for all three benchmarking exercises.
